# Media Discourse on Healthy Cities: Topic Modeling of South Korean News Articles (2004–2024)

**DOI:** 10.1007/s11524-026-01100-3

**Published:** 2026-06-29

**Authors:** Hye Su Jeong, Haejoo Chung

**Affiliations:** 1 BK21 FOUR R&E Center for Learning Health Systems, Korea University, 145 Anam-Ro, Seongbuk-Gu, Seoul, 02841 South Korea; 2 Division of Health Policy & Management, College of Health Science, Korea University, 145 Anam-Ro, Seongbuk-Gu, Seoul, 02841 South Korea; 3 Centre for Global Social Policy, University of Toronto, 700 University Ave, Toronto, ON M5G 1X6 Canada

**Keywords:** Cities, Public health, Media discourse, Urban health, Data mining, BIGKinds

## Abstract

This study aimed to analyze healthy and sustainable city-related news articles in South Korea to identify key themes in media discourse and their temporal changes, and to examine their alignment with the UN’s Sustainable Development Goals (SDGs). This was a retrospective observational study using text mining and topic modeling of news articles from a nationwide news database spanning 2004–2024. News articles (*n* = 15,137) containing the term “healthy city” were collected from the BIGKinds news big data system. After data preprocessing, Latent Dirichlet Allocation topic modeling was applied to identify main themes. Topic temporal changes were analyzed through linear trend analysis, and topics were evaluated for SDG relevance. Ten main topics were identified through topic modeling: eight related to SDG 11 (Sustainable Cities and Communities) and two to SDG 16 (Peace, Justice, and Strong Institutions). Topics showing significant increase over time included urban planning, policy governance, and policy evaluation. Interest decreased significantly in region-specific development, physical activity infrastructure, and local tourism. Notable gaps were found in addressing WHO-emphasized SDGs 2, 6, 12, and 13. South Korean media discourse on healthy cities has shifted from physical infrastructure development to urban planning and governance. While strongly aligned with SDG 11, findings suggest the need for a more balanced approach that addresses gaps in environmental sustainability while maintaining effective governance.

**Supplementary Information:**

The online version contains supplementary material available at https://doi.org/10.1007/s11524-026-01100-3.

## Introduction

According to the UN, approximately 67% of the global population will reside in urban areas by 2050 [1]. Rapid urban population growth has been accompanied by public health challenges including urban poverty, social inequality, and the spread of infectious diseases [2], as well as increased mental health issues [3]. In response, the WHO proposed the European Healthy Cities Project in 1986, which presented an integrated approach to health promotion by prioritizing health in urban political and social decision-making [4]. A healthy city creates and improves environments that enable residents to support each other and develop to their maximum potential [5]. The concept of healthy cities is linked to the UN’s Sustainable Development Goals (SDGs); health-promotion efforts based on healthy cities contribute to various SDGs, though they are particularly closely linked to SDG 11, “Make cities and human settlements inclusive, safe, resilient and sustainable” [6].

There is growing recognition among urban planners and policy makers that sustainability should be a paramount objective in urban planning and improvement [7]. Although achieving the SDGs requires an integrated and systematic global approach, tailored approaches that align with the realities, capacities, and development levels of individual nations and regions are also needed. The SDGs must be translated into tangible and specific actions, necessitating strategies that consider local characteristics [8].

Healthy city research has been conducted from various perspectives, including comprehensive reviews of the concept and global development status of healthy cities [9, 10], studies developing healthy city indicators [11], and systematic analyses of essential health risk factors for improving urban health [12]. The relationship between urban environments and health has also been studied from multiple angles. Khomenko et al. [13] analyzed the impact of urban and transport planning on health through a case study of Vienna, while Ali et al. [14] proposed an urban green space planning model targeting improved health among older adults. Mueller et al. [15] suggested the “superblock model” for transforming urban design in a health-promoting way. However, most studies have adopted an expert-centric approach; healthy city research that analyzes how the broader public engages with healthy city concepts—through channels such as media discourse—is relatively scarce.

Media coverage plays a crucial role in shaping public health policy and societal understanding of health issues [16], by communicating health risks to the public, which in turn influences policy prioritization [17, 18]. News media do not merely reflect public opinion; they actively frame issues through editorial decisions, journalistic conventions, and media agendas, thereby shaping how audiences perceive and prioritize health topics. For example, during COVID-19, news media dissemination of public health campaigns positively influenced individual hygiene habits and social distancing [19]. In addition, media coverage of e-cigarettes has impacted public perceptions and policy formation [20]. Analyzing media discourse thus provides valuable insights into how health concepts are communicated and framed in public discussion, even as it reflects media agendas rather than direct public opinion.

Media coverage primarily consists of unstructured natural language data; text mining can efficiently analyze and identify meaningful patterns in these data [21]. Topic modeling is a powerful technology for finding relationships between data and text [22, 23] and is actively used in urban studies; for example, to analyze smart city research trends using academic papers [24]; to present a smart city framework using various text data including scientific publications, news, and social media [25]; and to examine urban residents’ perceptions using media reports [26, 27].

However, research analyzing healthy-city-related media discourse is lacking, and there is a need for studies considering regional contexts. Addressing these gaps, this study aims to identify the main themes of media discourse on healthy cities and their evolution by conducting topic modeling on healthy-city-related media coverage in South Korea, and to link the derived topics with the SDGs to identify which SDGs are receiving relatively more and less healthy-city-related media attention.

## Methods

This study utilized BIGKinds (http://www.kinds.or.kr/), a news big-data system operated by the Korea Press Foundation that integrates news content from 54 South Korean media outlets, including general daily, financial, and local newspapers from 1990 to the present [28].

### Search Term and Preprocessing

Articles containing the search term “healthy city” (건강도시) in Korean were collected from January 2004 to July 2024, corresponding to the period when healthy cities were formally introduced in South Korea with four cities joining the Alliance for Healthy Cities [29]. This single search term was chosen because it is the officially adopted Korean term for the WHO Healthy Cities framework and is consistently used in policy documents, municipal designations, and media reporting in South Korea. From the extracted metadata, news identifier, title, publication date, and keywords were selected for analysis.

The keywords were generated through BIGKinds’ topic rank algorithm [28], a patented keyword extraction system (Korean Patent No. 1010570750000) that performs co-occurrence analysis on full article text, constructs semantic networks through word clustering, and extracts keywords based on both their frequency and contextual importance within each article. The system incorporates the Bareun morphological analyzer for Korean-language processing and uses BERT-based (Bidirectional Encoder Representations from Transformers) named entity recognition to identify persons, organizations, and locations.

The initial search resulted in 20,397 articles, reduced to 15,348 after removing duplicates. Subsequently, articles unrelated to healthy cities (e.g., articles with personnel changes [인사] in the title) were excluded. Finally, 15,137 articles remained for analysis (see Supplementary Fig. 1 for a PRISMA-style flow diagram of the article selection process). The data cleaning process involved the following procedures: removal of unnecessary characters (punctuation, control characters) from keyword data; removal of English text (as some articles contained auto-translated segments embedded in metadata); removal of Chinese characters (which occasionally appeared in proper nouns or historical references); removal of words starting with numbers or special characters; and removal of stop words (“healthy city,” “health,” “city,” “South Korea”).

Finally, a text normalization process was conducted to integrate keywords with semantic similarities into representative terms using a comprehensive normalization dictionary containing 10,608 term mappings. For example, the Korean terms “노인” (elderly), “고령자” (aged), “시니어” (senior), “어르신” (elder), and “은퇴세대” (silver generation) were consolidated into the representative term “고령자” (older adults). Similarly, “돌봄” (care) and “돌봄서비스” (care services) were integrated into “돌봄” (care), and “힐링시티” (healing city) and “힐링타운” (healing town) were consolidated into “힐링도시” (healing city).

### Term Frequency and Term Frequency-Inverse Document Frequency

Term frequency (TF) and term frequency-inverse document frequency (TF-IDF) metrics were utilized to quantify both term frequency and its relative importance within the document corpus, where IDF assigns lower weights to common terms and higher weights to rare terms [30]. TF-IDF values were calculated using the bind_tf_idf function from the tidytext package in R software.

### Latent Dirichlet Allocation Topic Modeling

Topic modeling is an unsupervised machine learning technique used to extract latent thematic structures from large-scale unstructured document collections [31]. Latent Dirichlet Allocation (LDA), proposed by Blei et al. [22], is one of the most extensively applied topic modeling algorithms [23], where each document is represented as a probabilistic distribution of latent topics sampled from a Dirichlet distribution [22]. In this study, modeling was performed using the LDA function of the topicmodels package in R software, with the Variational Expectation-Maximization (VEM) algorithm as the estimation method. The alpha parameter (Dirichlet prior for document-topic distributions) was set to the default value of 50/k, and the topic-word distribution parameter was estimated during the VEM optimization process. The random seed was set to 1234 for reproducibility (see Supplementary Table 1 for complete parameter specifications).

When executing topic modeling, determining the number of topics, k, is a prerequisite. In this study, four evaluation metrics were used to derive the optimal number of topics. The Cao et al. [32] and Arun et al. [33] metrics are interpreted as having higher model suitability with lower values, whereas the Griffiths [34] and Deveaud et al. [35] metrics indicate higher model suitability with higher values. The calculation of these metrics was performed using the FindTopicsNumber function from the ldatuning package in R software, with Gibbs sampling (seed = 1234) used to evaluate k values ranging from 2–20. In this study, the inflection point where no clear gain in model performance was observed with additional increases in the number of topics was identified at k = 10, which was thus adopted as the final number of topics (Supplementary Fig. 2).

The topics derived through topic modeling were evaluated for their relevance to the SDGs, focusing on SDGs 2 (Zero Hunger), 6 (Clean Water and Sanitation), 11 (Sustainable Cities and Communities), 12 (Responsible Consumption and Production), 13 (Climate Action), and 16 (Peace, Justice and Strong Institutions). These are emphasized by the WHO in relation to healthy cities [6], even though they do not directly mention health as in SDG 3 (Good Health and Well-Being). In this process, one researcher (H.S.J.) conducted the initial mapping by systematically comparing each topic’s top-weighted keywords against the specific targets and indicators listed under each SDG. Cases where the topic–SDG connection was ambiguous were then discussed between both authors to reach a consensus.

Topic prevalence indicates how much each document is associated with a specific topic [36]. LDA assumes that each document is composed of multiple topics; their ratio is modeled as being drawn from a Dirichlet distribution [22]. The parameter γ of this distribution represents the topic distribution for each document. In this study, the γ values for documents in the corpus were averaged to quantify the proportion of each topic in the entire document collection.

Changes in average γ over time demonstrate shifts in media interest for each topic. In this study, a linear trend analysis was performed on the yearly average γ for each topic throughout the research period [34]. Topics with positive (negative) slopes were classified as hot (cold) topics. These trends were considered statistically significant at p < 0.05.

## Results

**Fig. 1:**
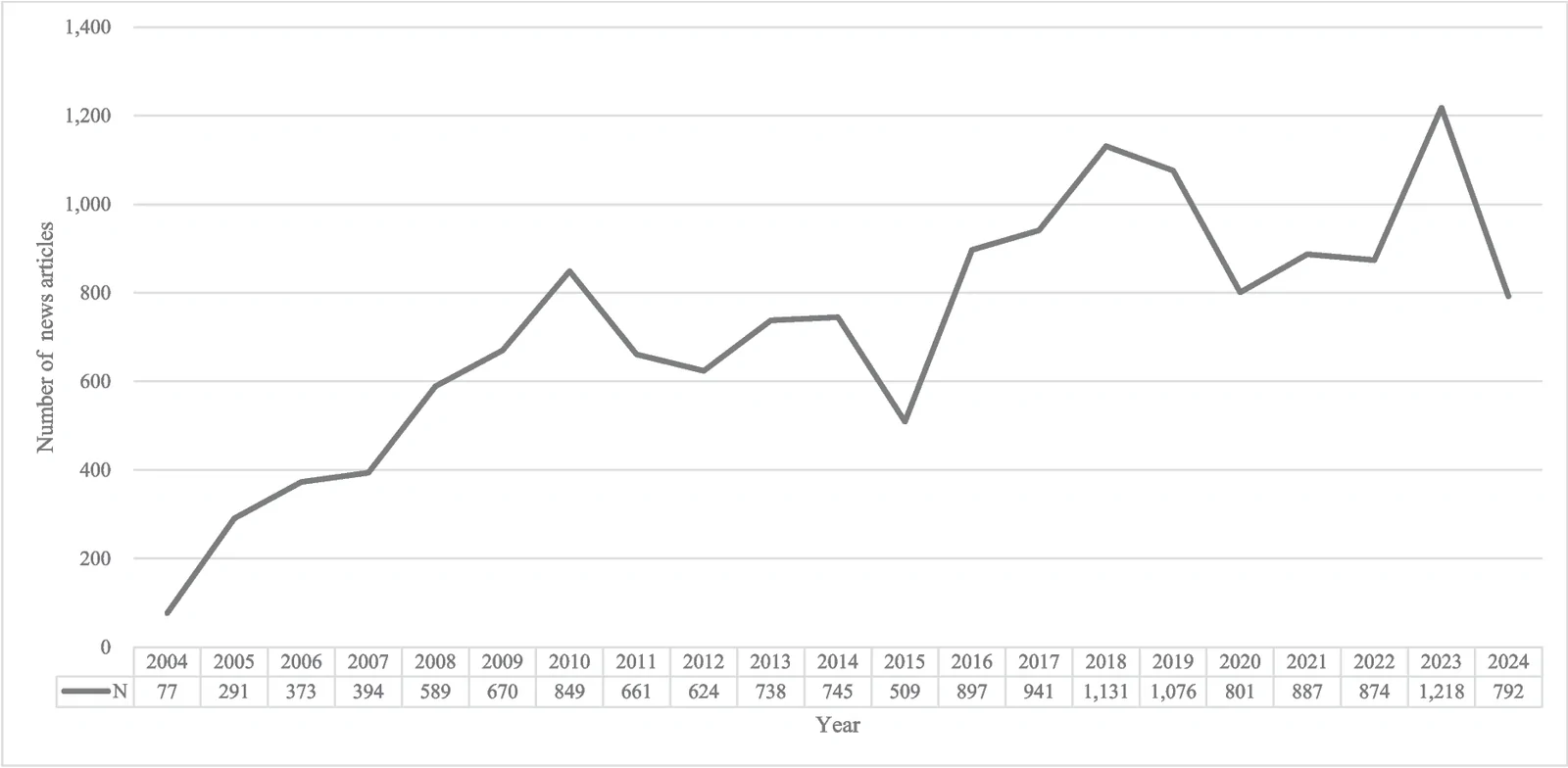
Trend in news article publications related to healthy cities in South Korea (2004–2024)

Figure 1 presents the frequency of healthy-city-related articles published from 2004 to 2024, showing an overall upward trend. Before 2007, there were fewer than 500 articles annually, but the number gradually increased thereafter. Notably, in 2018–2019 and 2023, the annual publication number exceeded 1,000. Hence, South Korean media interest in healthy cities appears to have grown continuously.

Table 1 presents the top 30 keywords related to healthy cities in media discourse, identified through TF and TF-IDF analysis. TF analysis showed the most frequent words were “planning,” “support,” “local residents,” “mayor,” and “environment,” indicating healthy city media discourse’s association with urban planning, community support, and administrative leadership. The TF-IDF results highlighted the importance of specific regions and places (e.g., “Jincheon Central Market,” “Yangsan Fire Station,” “Gyeongju”), reflecting the diverse implementation of healthy city activities according to local characteristics.

**Table 1:** Top 30 keywords in healthy city news articles: comparison of term frequency (TF) and term frequency-inverse document frequency (TF-IDF) analysis

	TF Analysis		TF-IDF Analysis	
	Keywords	Frequency	Keywords	TF-IDF Score
1	Planning	14,930	Jincheon Central Market	2.04
2	Support	12,887	Yangsan Fire Station	1.69
3	Local residents	12,441	Gyeongju	1.68
4	Mayor	9448	Lee Kwang-jun	1.66
5	Environment	8705	Geumcheongu	1.56
6	Policy	8447	Dalseogu Senior Welfare Center	1.53
7	Culture	8400	Yangsan Citizens	1.53
8	Participation	8251	Jincheon	1.46
9	Education	7780	Choi Jae-gu	1.44
10	Healthcare	7398	National Unification Advisory Council	1.41
11	Living conditions	7174	Cooperation Ceremony	1.26
12	Development	6792	Changwon Forum	1.26
13	Public health	6755	Asan	1.25
14	Well-being	6626	Slow Food	1.25
15	Revitalization	6565	Symposium	1.19
16	Welfare	6299	Gumi	1.19
17	Industry	6265	Wonju Future Healthy City	1.19
18	Management	6230	Family Sports Event	1.19
19	Evaluation	6197	Hong Ju-seong	1.18
20	Efforts	6075	Sports Knowledge	1.17
21	Seoul	6000	Kang Soo-myung	1.16
22	Wonju	5970	Gunsan	1.16
23	Program	5938	Lee Gye-jin	1.15
24	Infrastructure	5906	Autoimmunity	1.13
25	Elderly	5738	Baek Jong-su	1.13
26	Safety	5670	Activity Group	1.13
27	Prevention	5495	Hwang Seon-bong	1.12
28	Intention	5432	Yuseonggu	1.10
29	Smoking cessation	5419	Changwon	1.09
30	Tourism	5405	Gimje	1.09

LDA topic modeling revealed 10 themes related to healthy city during the study period, which are classified according to the SDGs (Table 2). SDG 11 (Sustainable Cities and Communities) includes eight topics: Topic 1 focuses on health promotion activities (e.g., “smoking cessation,” “program,” “physical activity”). Topic 2 emphasizes region-specific development (e.g., “wonju,” “plan,” “enterprise”). Topic 3 focuses on infrastructure development for promoting physical activity (e.g., “physical activities,” “bicycle,” “facility”). Topic 4 is linked to public health services (e.g., “medical,” “public health,” “prevention”). Topic 5 reflects discussions on urban planning (e.g., “plan,” “industry,” “culture”). Topic 7 concentrates on local cultural tourism and economic revitalization (e.g., “festival,” “oriental medicine,” “tourism”). Topic 9 deals with community welfare (e.g., “support,” “education,” “welfare”). Topic 10 focuses on evaluation of healthy city policy (e.g., “assessment,” “policy,” “excellence”).

**Table 2:** Latent Dirichlet allocation topic modeling of healthy city news articles: topics and associated keywords

	Label	Words
*SDG 11: Sustainable Cities and Communities*		
Topic 1	Health Promotion Activities	Smoking cessation, Participation, Residents, Event, Program, Smoking, Physical activity, Public health center, Practice, Competition
Topic 2	Regional Specialized Development	Wonju, Plan, Enterprise, Industry, Attraction, Support, Development, Medical, Construction, Tourism
Topic 3	Physical Activity Promotion Infrastructure	Physical activities, Competition, Bicycle, Facility, Sports, Daily life, Ulsan, Jeju Island, Plan, Wonju
Topic 4	Public Health Services	Medical, Public health, Support, Prevention, Management, Public health center, Services, Residents, Dementia, Disease
Topic 5	Urban Planning	Plan, Chungju, Industry, Support, Market, Culture, Future, Environment, Revitalization, Daily life
Topic 7	Local Cultural Tourism and Economic Revitalization	Jecheon, Festival, Oriental medicine, Plan, Tourism, Jincheon, Event, Experience, Jinan, World
Topic 9	Community Welfare	Jincheon, Support, Education, Popularly elected, County residents, Plan, Welfare, County governor, Happiness, City mayor
Topic 10	Healthy City Policy Evaluation	Assessment, Policy, Excellence, Enrollment, Environment, Alliance for Healthy Cities, Public health, Plan, Residents, Effort
*SDG 16: Peace, Justice and Strong Institutions*		
Topic 6	Policy and Governance	Seoul, Residents, Support, Plan, Policy, City mayor, Culture, COVID-19, District mayor, Welfare
Topic 8	Local Elections and Regional Development Policies	Candidate, Pledge, Geumsan, Market, Election, Ginseng, Culture, Support, Tourism, Development

SDG 16 (Peace, Justice and Strong Institutions) includes two topics: Topic 6 addresses policy and governance issues (e.g., “policy,” “plan,” “city mayor”). Topic 8 focuses on local elections and regional development policies (e.g., “candidate,” “pledge,” “election”). We did not find topics directly related to the main SDGs emphasized by the WHO in the context of healthy cities (e.g., SDGs 2, 6, 12, and 13).

**Fig. 2:**
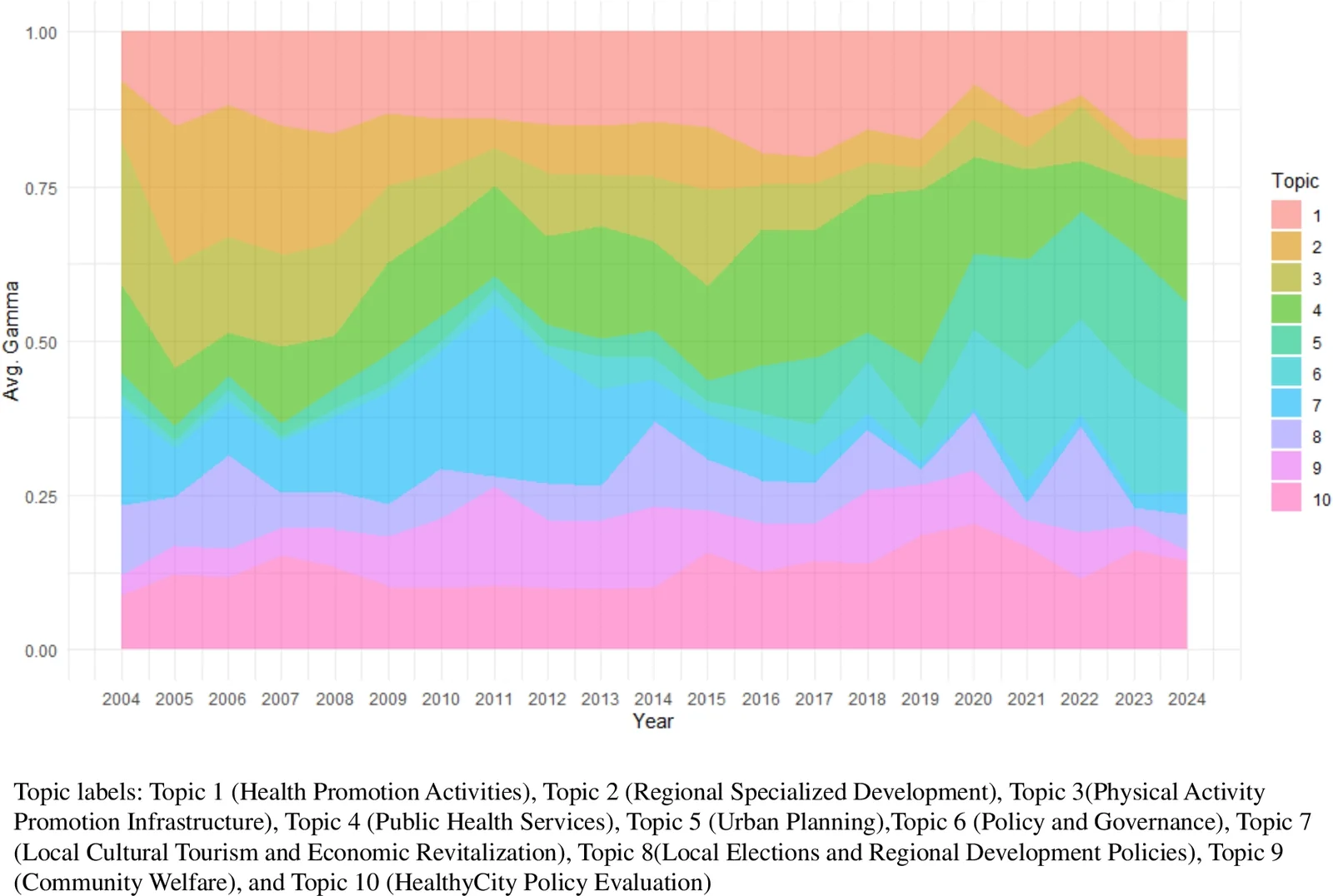
Trends of topic prevalence in healthy city news articles in South Korea (2004–2024)

Figure 2 shows temporal changes in the prevalence of healthy city-related topics. Topic 1 (health promotion activities) and Topic 10 (healthy city policy evaluation) appeared frequently throughout the period. Compared to the early stages in 2004, the proportions of Topic 5 (urban planning) and Topic 6 (policy and governance) significantly increased in recent years, particularly since 2020. By contrast, Topic 2 (region-specific development), Topic 3 (infrastructure development for physical activity), and Topic 7 (local cultural tourism and economic revitalization) showed a gradual decrease.

**Fig. 3:**
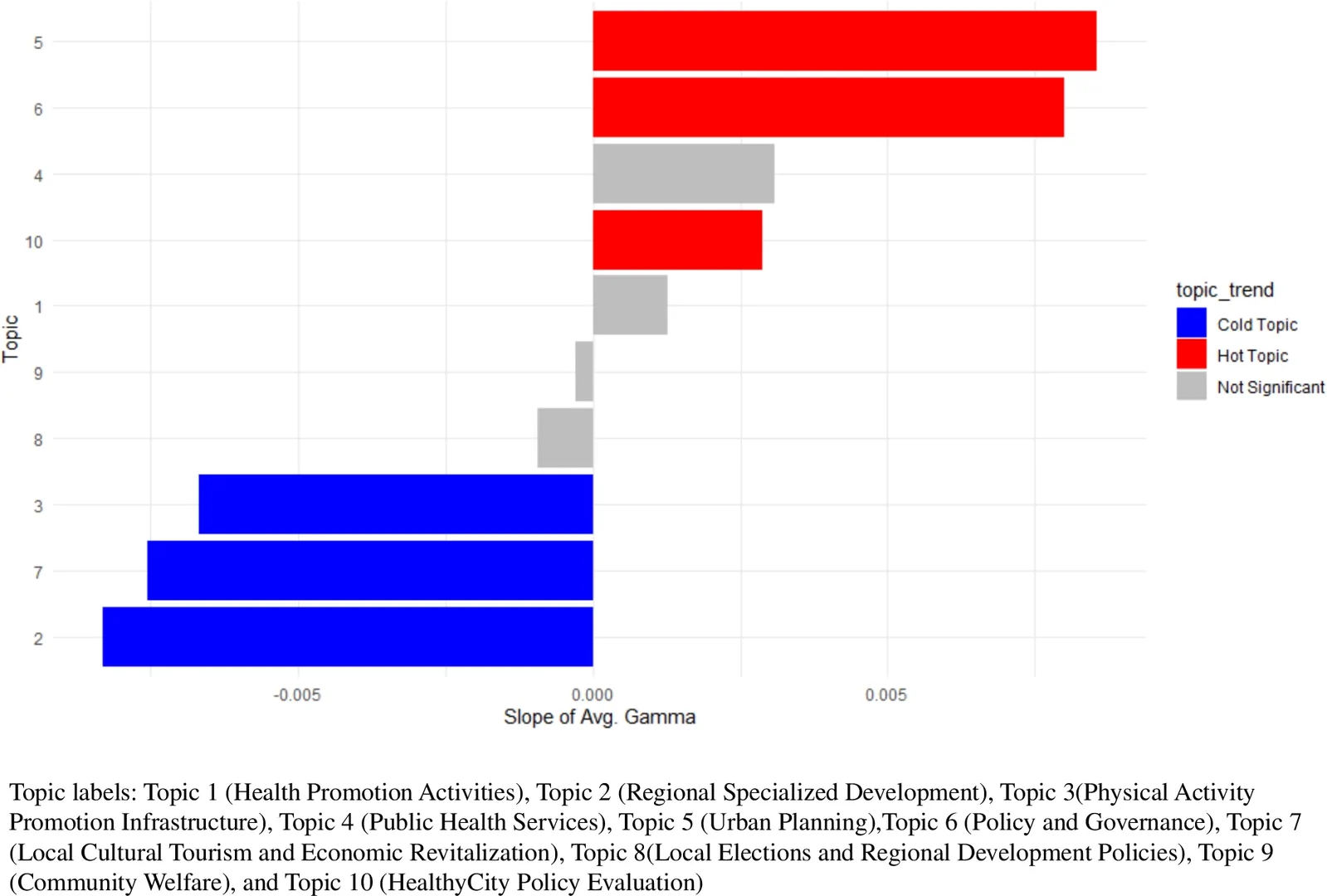
Hot and cold topics in healthy city news articles in South Korea (2004–2024)

Figure 3 shows the classification of derived topics into hot and cold topics based on their change trends. Topics 5, 6, and 10 are hot topics showing statistically significant upward trends, with Topic 5 exhibiting the steepest positive slope. Conversely, Topics 2, 3, and 7 are cold topics, demonstrating statistically significant downward trends, with Topic 2 showing the steepest negative slope. Hence, media interest in urban planning (Topic 5) has increased most significantly, whereas interest in region-specific development (Topic 2) has shown the biggest decrease.

## Discussion

This study systematically analyzed healthy-city-related articles published in South Korean media from 2004 to 2024 using LDA-based topic modeling to identify main themes of media discourse and their alignment with SDGs.

There was an overall upward trend in the frequency of healthy-city articles from 2004 to 2024, with annual publications exceeding 1,000 in some recent years. This trend aligns with national developments, particularly the establishment of Article 6-5 of the National Health Promotion Act in 2021, which provided a legal basis for healthy-city creation [37, 38]. A temporary decrease in publications was observed during 2020–2022: annual articles dropped from 1,076 in 2019 to 801 in 2020 and remained below the pre-pandemic level through 2022 (874 articles), before rebounding to 1,218 in 2023. This pattern likely reflected COVID-19’s disruption of routine healthy city activities rather than a fundamental shift in media attention.

TF and TF-IDF analyses of keywords suggest that media discourse on healthy cities in South Korea has multilayered characteristics, including regional diversity. TF analysis highlighted keywords such as “planning,” “support,” “local residents,” and “mayor,” while TF-IDF analysis emphasized specific regions and places, reflecting interest in both top-down and bottom-up approaches. This integrated approach is supported by various studies: Goel et al. [39] emphasized the necessity of combining strategic thinking with citizen participation in smart cities, while Yi et al. [40] and Wang et al. [41] demonstrated the importance of region-specific approaches in urban development. These findings indicate media interest in comprehensive approaches that integrate stakeholder participation with regional characteristics.

LDA topic modeling resulted in 10 major topics, with 8 topics related to SDG 11 (Sustainable Cities and Communities). Among these, Topic 1 (health promotion activities), Topic 3 (physical activity promotion infrastructure), Topic 4 (public health services), and Topic 9 (community welfare) were related to health and welfare. This reflects the close relationship between cities and health, which has been researched since industrialization emerged in the nineteenth century [42]. Modern healthy cities aim to promote overall health and reduce health inequalities through managing non-communicable diseases, creating pedestrian-friendly environments, and ensuring safety [43]. These health-related and welfare-related topics reflect modern urban health elements, aligning with the concept of Health in All Policies (HiAP) [44]. The importance of establishing common concepts between health and urban experts is increasingly emphasized [45], as evidenced by Dutch research showing that both fields’ experts prioritize quality space and exercise-conducive environments [46].

Topics related to regional development were identified as Topic 2 (regional-specialized development) and Topic 7 (local-cultural tourism and economic revitalization). These topics reflect the importance of regional context in achieving the SDGs, requiring consideration of regional specificities [47, 48]. This is supported by studies from China and Italy, which showed that SDG emphasis varies by regional economic and territorial characteristics [49, 50]. Similarly, media discourse in South Korea shows interest in regional development through region-specific industries, festivals, and tourism, suggesting the need for customized approaches in healthy-city policies.

Topics related to urban planning and policy evaluation were identified as Topic 5 (urban planning) and Topic 10 (healthy-city policy evaluation). These topics reflect the importance of considering the healthy-city concept from the urban-planning stage and continuously evaluating its effectiveness. COVID-19 has highlighted the need to consider public health during urban design, from immediate physical-distancing measures to long-term stakeholder awareness [51]. Evaluation of healthy-city policies is essential for urban sustainability and SDGs [52], where health impact assessment (HIA) can effectively support the HiAP approach through comprehensive consideration of health economics and stakeholder participation [53]. The emergence of these topics suggests that South Korea’s healthy city policies recognize the importance of systematic management from planning to evaluation.

Topics related to SDG 16 (Peace, Justice and Strong Institutions) were identified as Topic 6 (policy and governance) and Topic 8 (local elections and regional development policies). The importance of governance is confirmed at both national and city levels. A study of 101 countries revealed that governance indirectly positively affects individual health by improving income and healthcare quality [54]. Local governments emphasize networked forms of governance through stakeholder collaboration to solve complex urban problems [55], which influences local elections and policy-making processes [56]. For instance, urban planning pledges and budgets in local elections were associated with higher vote shares [57]. The emergence of these topics indicates that media discourse on healthy cities in South Korea recognizes the importance of effective local governance and participatory policy-making.

In summary, the derived topics primarily align with SDGs 11 and 16, corresponding with the goal to “make cities and human settlements inclusive, safe, resilient and sustainable” [58]. This alignment pattern is similar to smart city research, which also focuses on SDG 11 while identifying SDG 2 as needing additional attention [59], reflecting the shared goal of sustainable urban development.

Notably, we did not identify topics directly related to other SDGs emphasized by the WHO in the context of healthy cities, such as SDGs 2, 6, 12, and 13. This reveals a gap between South Korean society’s media discourse on healthy cities and these SDGs, suggesting the need for additional policy consideration in areas such as food security, water and sanitation, sustainable consumption and production, and climate change responses.

Topic trend analysis revealed that Topic 5 (urban planning), Topic 6 (policy and governance), and Topic 10 (healthy city policy evaluation) showed statistically significant increases in media interest, while Topic 2 (region-specific development), Topic 3 (infrastructure development for physical activity), and Topic 7 (local cultural tourism and economic revitalization) showed statistically significant decreases. This partially aligns with the 40-year trend in healthy city research, which shows expanded interest in urban design and resident health factors [60]. The emergence of urban planning and policy evaluation as hot topics aligns with previous research emphasizing the complex interaction between urban environments and health [61]. However, unlike Jia et al.’s [60] finding of active physical activity research, our study showed decreased media interest in this area, possibly due to South Korea’s established urban infrastructure. This shift from foundation-building to policy and evaluation aligns with current trends in South Korean healthy city research [62], suggesting the need to balance continued focus on urban planning while stimulating interest in physical activity and regional development.

It should be noted that the 54 news outlets included in BIGKinds encompass national dailies, financial newspapers, and local/regional papers. Local newspapers naturally focus more on their respective regions, which likely contributes to the prominence of specific place names in the TF-IDF results. This regional variation in coverage patterns means that certain regions may receive disproportionate media attention based on the editorial focus of their local outlets. However, this regional diversity also reflects an important aspect of healthy city implementation in South Korea, which is fundamentally a locally-driven initiative. In addition, the 54 outlets included in BIGKinds encompass media across the political spectrum, from outlets generally considered conservative (e.g., Chosun Ilbo, JoongAng Ilbo, Dong-A Ilbo, The Korea Economic Daily) to progressive (e.g., Hankyoreh, Kyunghyang Shinmun). While healthy city discourse—being primarily a local governance and public health initiative—is less susceptible to strong ideological framing than partisan policy issues, editorial orientation was not separately analyzed; future research could compare topic distributions across outlets of different editorial orientations.

The study has the following limitations: First, owing to data source limitations, this study only analyzed newspaper articles, excluding media discourse from other sources such as social media and limiting both regional analysis and result generalizability. Second, the search strategy relied solely on the Korean term “건강도시” (healthy city), which may exclude relevant articles that discuss healthy city concepts—such as urban walkability, green spaces, or air quality—without using this exact term. Third, this study used keywords pre-extracted by BIGKinds’ topic rank algorithm rather than full article text; while this provides a consistent and standardized basis for analysis, it introduces a preprocessing layer that may compress nuanced content, potentially favoring commonly co-occurring terms and overrepresenting named entities. Fourth, the linear trend analysis used to classify hot and cold topics may oversimplify more complex temporal patterns, such as the non-linear dip observed during 2020–2022 due to COVID-19; future research could employ dynamic topic modeling [63] or piecewise regression to better capture these patterns. Fifth, researchers’ subjective judgments may intervene in the preprocessing of topic modeling and topic labeling. Sixth, the current study design does not permit systematic regional comparisons; future research could conduct separate topic models by region or newspaper type to examine geographic variation in healthy city discourse. Future research should address these limitations through diverse data sources, broader search strategies, and dynamic topic modeling techniques.

Nonetheless, the significance of this study is as follows: First, it conducted a systematic analysis of healthy-city-related media discourse in South Korea using topic modeling of 20-year news article data. Second, by analyzing links between derived topics and the SDGs, it identified alignment patterns and gaps with the sustainable development agenda. Third, it tracked temporal changes in media discourse, providing insights for future policy directions.

## Conclusion

This study analyzed media discourse on healthy cities in South Korea through topic modeling of news articles spanning two decades. The findings reveal a significant evolution in media interest, shifting from physical infrastructure towards urban planning and policy governance. While the identified topics strongly align with SDG 11, gaps exist in addressing other WHO-emphasized SDGs related to food security, water management, and climate action. These findings suggest the need for a balanced approach that maintains focus on governance while addressing environmental sustainability, indicating future directions for healthy city policies in South Korea.

## Supplementary Information

Below is the link to the electronic supplementary material.ESM 1(DOCX 122 KB)

## Data Availability

The data used in this study are available through BIGKinds (https://www.bigkinds.or.kr/).
