# Supplementary material for: Media Discourse on Healthy Cities: Topic Modeling of South Korean News Articles (2004–2024)
Source: J Urban Health. 2026 Jun 29;103(4):833–44. doi: 10.1007/s11524-026-01100-3 (PMC13525135; doi:10.1007/s11524-026-01100-3)
Supplement: Supplementary file 1 [file 11524_2026_1100_MOESM1_ESM.docx]

**Supplementary Figure 1. Flow Diagram of Article Selection Process**

**Supplementary Figure 2. Evaluation of Optimal Number of Topics for Latent Dirichlet Allocation Model**

**Supplementary Table 1. Latent Dirichlet Allocation Model Parameter Specifications**

| **Parameter** | **Value** |
| --- | --- |
| Estimation method | Variational Expectation-Maximization (VEM) |
| Number of topics (k) | 10 |
| Alpha (α, Dirichlet prior for document-topic distributions) | 50/k = 5.0 (default) |
| Beta/Delta (topic-word distribution parameter) | Estimated by model during VEM optimization |
| EM maximum iterations | 1,000 (default) |
| Variational inference maximum iterations | 500 (default) |
| Random seed | 1234 |
| R package | topicmodels |
| Topic number optimization method | FindTopicsNumber (ldatuning package) |
| Optimization estimation method | Gibbs sampling |
| Optimization range | k = 2 to 20 |
| Optimization metrics | CaoJuan2009, Arun2010, Griffiths2004, Deveaud2014 |
| Optimization random seed | 1234 |
